# Concomitant spontaneous spinal and posterior fossa subdural hematoma in an 11-year-old child with aplastic anemia: a case report and review of literature

**DOI:** 10.1007/s00381-022-05584-7

**Published:** 2022-06-21

**Authors:** Ali M. Abou-Madawi, Mohamed K. Elkazaz, Alaa El-Din Saad Abdelhamid

**Affiliations:** 1 Neurosurgery Department, Faculty of Medicine, Suez Canal University Teaching Hospital, Suez Canal University, Ismailia, Egypt; 2 Hematology, Clinical and Chemical Pathology Department, Faculty of Medicine, Suez Canal University Teaching Hospital, Suez Canal University, Ismailia, Egypt

**Keywords:** Subdural hematoma, Aplastic anemia, Spontaneous, Concomitant

## Abstract

**Purpose:**

The current article describes an 11-year-old male who has aplastic anemia with an extremely rare condition, that is, concomitant posterior fossa SDH and spinal SDH.

**Methods:**

This is a case report and review of literature.

**Case presentation:**

This case presents an 11-year-old male known to have aplastic anemia complained of neck and back pain, headache, and persistent vomiting for 3 days. He had no history of head or spine trauma at all. His parents are relatives “positive consanguinity,” and his sister suffers from aplastic anemia. Clinical examination revealed severe pallor at the time of presentation, with no neurologic or locomotor deficit and positive Kernig’s sign.

**Conclusion:**

Patients with aplastic anemia or any bleeding disorder conditions should be investigated thoroughly if symptoms denoted a CNS pathology. Concomitant cranial and spinal SDH rarely occurs, and more studies are advocated to be structured to investigate the specific pathophysiology and etiologies of this condition.

## Introduction

Spontaneous posterior fossa and spinal subdural hematomas (SDH) are extremely uncommon pathologies along with poorly elucidated pathogenesis. In addition, their concomitant occurrence is extremely rare [1, 2]. Spinal SDH comprises 6.5% of all spinal hematomas [3], and the incidence of posterior fossa SDH is 0.01% [4]. Moreover, recorded cases with concomitant spontaneous occurrence of both posterior fossa and spinal SDH with aplastic anemia are extremely rare [5–9]. SDH commonly occurs due to various factors, including vascular malformations, senile brain atrophy, hematological disorders, or iatrogenic factors such drugs side effects [8, 10, 11]. Aplastic anemia is a rare disorder, where patients present with bone marrow suppression, resulting in pancytopenia [12]. The definitive etiology is still unclear, but it is thought to be an immune-based disease that is triggered by some genetic predispositions. Bleeding tendency is one of the hallmarks of this disease because of thrombocytopenia [13]. The present article describes an 11-year-old male who has aplastic anemia with an extremely rare condition, that is, concomitant posterior fossa SDH and spinal SDH.

## Case presentation

An 11-year-old boy known to have aplastic anemia presented to our service complaining of headache and persistent vomiting and neck and back pain for 3 days. He had no history of head or spine trauma at all. His parents are relatives “positive consanguinity,” and his sister also suffers from aplastic anemia. Clinical examination at the time of presentation revealed severe pallor with petechial rashes all over the body, with no definite weakness or neurologic or locomotor deficit. Signs of meningeal irritation including neck rigidity and Kernig’s sign were positive. His blood picture indicated pancytopenia (HB = 8 g/dl, WBC = 1.59 10^3/uL, and platelets count = 19.5 10^3/uL). PT, PTT, and other liver function tests were normal. D-dimer and hepatitis B and C serological tests were negative. Bone marrow biopsy presented markedly hypocellular bone marrow with 20% cellularity. Abdominal ultrasonic scan excluded chronic liver disease and hypersplenism.

**Fig. 1:**
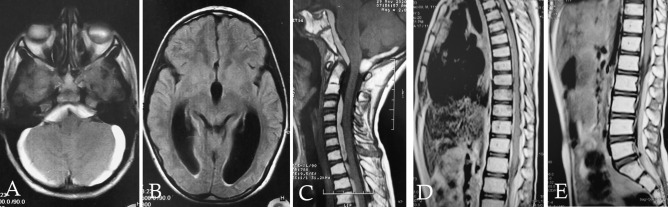
Initial MRI of the whole craniospinal axis at the time of presentation. **A** Posterior fossa T1W axial image showing posterior fossa and peri-mesencephalic SDH, **B** supratentorial T1W axial image showing supratentorial obstructive hydrocephalus, **C** T1W sagittal image showing SDH extending anteriorly opposite the whole clivus and posteriorly opposite the surface of both cerebellar hemispheres and extending anteriorly to the whole cervical spinal cord, **D** thoracic spine T1W sagittal image showing SDH extending anteriorly to the thoracic spinal cord, and **E** lumbosacral T1W sagittal image showing SDH extending posteriorly to the cauda equina down to the level of the lower end of the sacrum and associated with cauda equina compression

A CT scan of the brain was ordered on emergency bases and showed dilation of the supratentorial ventricles with acute SDH extending over both cerebellar hemispheres and retroclival space. Brain MRI showed posterior fossa and peri-mesencephalic SDH extending anteriorly opposite the whole clivus and posteriorly opposite the surface of both cerebellar hemispheres and associated supratentorial obstructive hydrocephalus. Whole spine MRI showed SDH extending from the lower border of C2 vertebra along the whole spine and down to the level of the lower end of the sacrum associated with spinal cord compression (Fig. 1). A multidisciplinary approach including neurosurgery, pediatric hematology, and clinical pathology team was involved for the management and decision-making of this extremely rare and challenging case.

**Fig. 2:**
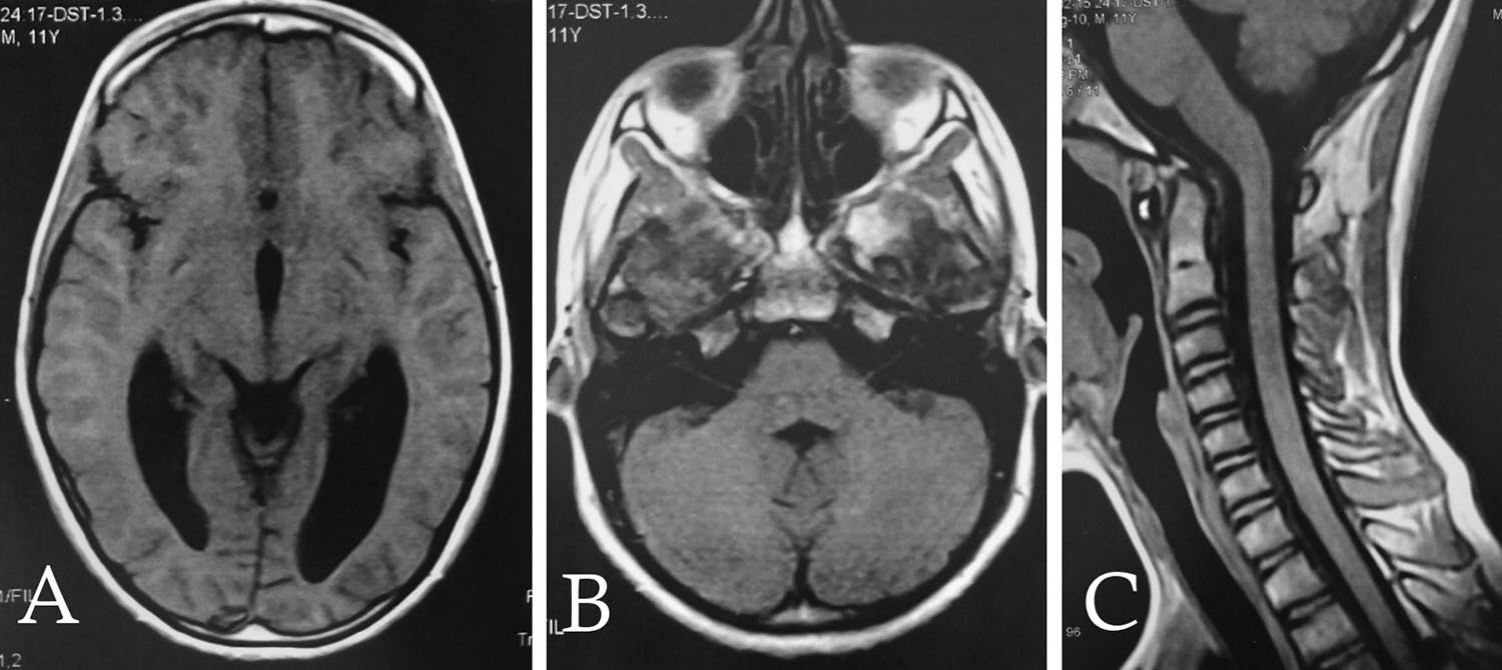
A 6-month follow-up MRI brain and cervical spine showing **A** marked regress of hydrocephalus, **B** complete resorption of the posterior fossa SDH, and **C** complete resorption of the clival, retro-cerebellar, and cervical SDH

After discussion of the pros and cons of different therapeutic options, it has been decided to put the patient under strict observation with conservatives and supporting measures in the ICU. Patient received fluids, steroids, diuretic, and paracetamol infusion. The medical management of aplastic anemia included thrombopoietin receptor agonist TPO-RA; cyclosporin; folic acid; nutritional supplement for vitamins B1, B6, and B12; and Deferasirox. Serial clinical follow-ups were performed for the patient, and his symptoms and signs as well as his hematopoietic parameters showed gradual improvement. He has stayed in the ICU for 10 days, then was discharged to the inpatient ward for a week, then was discharged home from the hospital, and was scheduled for routine outpatient clinic visits. One month after the episode, the patient was clinically symptoms free. A brain and a whole spine MRI were ordered for the patient after 6 months, and they showed complete resolution of the SDH from the whole craniospinal axis with decreased ventricular size (Fig. 2).

## Discussion

Concomitant spinal and cranial SDH is a very rare and challenging clinical situation with quiet few cases having been reported in literature. To the best of our knowledge, only 50 cases have been published in literature including only 2 cases of anaplastic anemia [14]. The underlying predisposing factors in such condition could be trauma, vascular malformation, bleeding disorder due to antiplatelet or anticoagulant therapy or thrombocytopenia, bleeding tumor, lumbar puncture, alcohol abuse, or spontaneous [7, 14]. Reporting concomitant spontaneous spinal and cranial SDH in patients with aplastic anemia is very rare with only 2 cases having been reported in literature. In this report, we add to literature the third case (Table 1). The first case was reported in 2008, a 12-year-old male with posterior fossa and spine SDH. The patient was managed conservatively by platelet-rich plasma, mannitol, and steroids [6]. The second case was reported in 2017, a 14-year-old male with bi-frontal, posterior fossa, and spine SDH. The patient was also managed conservatively by multiple blood transfusions and received anti-thymocyte globulins [7].

**Table 1:** Data summary of reported cases in the literature

No.	Author	Year	Age/sex	Clinical presentation	Imaging data	Management	Outcome
1	Jain et al. [6]	2008	12/M	Headache and back pain for 3 days	Posterior fossa and C1 to S3 SDH	Conservative	Good
2	Satyarthee and Ahmad [7]	2017	14/M	Headache and back pain for 12 h	Bi-frontal, posterior fossa, cervical, and dorsal SDH	Conservative	Good
3	This case	2022	11/M	Headache and neck and back pain for 3 days	Posterior fossa and C2 to sacrum SDH	Conservative	Good

This case is a known child with history of aplastic anemia who presented with symptoms and signs related to the intracranial component associated with back pain and manifestations of severe aplastic anemia. After careful decision-making by multidisciplinary medical approach, he responded very well to conservative measures and became symptom free within one month and SDH completely resolved on a 6-month MRI.

The specific mechanism for developing the spinal component of the SDH is still unclear as there are no bridging veins in this site in contrast to cranial SDH [10]. There are proposed theories for this condition, and the most accepted one is the blood redistribution from cranial SDH to the spinal SDH, but still there is no proof for this theory. Chronic SDH has membranous boundaries unlike the acute SDH, when acute hemorrhage occurs on top of chronic SDH resulting in the membrane rupture and leakage of the content. Then, by the effect of gravity, SDH can dissect its way along spinal subdural space [8, 11, 15]. Another theory was proposed in patients with ventriculoperitoneal shunts, where low intracranial pressure can cause widening of the subdural space resulting in tears in the bridging veins and opening a tract for SDH to move to the spinal compartment [16]. Furthermore, Ichinose et al. [17] suggested that concomitant cranial and spinal SDH may occur separately as a result of double traumas to the head and the spine.

There are similar published cases with spontaneous concomitant spinal and cranial SDH. Broc-Haro et al. [18] reported a 44-year-old patient with progressive headaches and lumbar radiculopathy. His brain and spine imaging showed frontotemporal SDH and spinal SDH. The patient received supportive therapy for headaches and radicular pain for 2 weeks and showed signs of improvement, and no surgical intervention was required. In addition, Moon et al. [16] reported a 39-year-old patient who was admitted for spinal SDH, and on admission, she started to complain from headache and nausea. Brain CT showed SDH with midline shift that both patients had neither predisposing factor for the SDH nor any history of trauma. The cranial SDH was surgically evacuated due to progressive headaches and to guard against cerebellar herniation. The patient was booked for a later evacuation of the spinal SDH. However, her back and radicular pains improved spontaneously, and no further surgical intervention was required. Lecouvet et al. [11] reported a patient with progressive pain and lower limb weakness. His spine MRI showed spinal SDH. Then, a brain MRI was performed due to the patient’s complaint of severe headaches, and it showed a cortical metastasis with SDH. The brain mass and SDH were surgically evacuated, while the spinal SDH symptoms were well tolerated by the patient and showed spontaneous improvement. Jain et al. [6] conservatively managed their aplastic anemia patient with concomitant cranial and spinal SDH by mannitol, steroids, and platelet rich plasma as a result of the poor general condition and the absence of any neurological deficits, and he had an uneventful recovery course.

Reviewing the reported two children plus our case indicated that this condition occurs in children, who responded very well to conservative therapy and demonstrated good outcome. Consequently, conservative management to address symptoms and treat underlying pathology is recommended in patients with poor general condition and no or mild neurological deterioration. Needless to say, the definitive treatment of aplastic anemia is bone marrow transplantation.

Cranial and spinal SDH is considered neurosurgical emergency especially when rapid neurological deterioration occurs. However, in neurologically stable patients, spontaneous resolution occurs and has been documented [11, 19, 20]. Surgical evacuation is spared for rapidly deteriorating neurological conditions and mainly contributed to the cranial SDH in fear for cerebellar herniation [11, 16]. Spinal SDH can be surgically evacuated in cases of rapidly neurologic deterioration either by open surgery or by minimal invasive lumbar puncture [17]. Most cases of concomitant cranial and spinal SDH had a good recovery spontaneously [8, 11, 21, 22].

## Recommendations

Concomitant cranial and spinal SDH requires a multidisciplinary approach, and in patients with predisposing factors, neural axis imaging is required when patients complain of both cranial and spine symptoms. Conservative management involving correcting the cause of bleeding disorder and symptoms relieving agents still has the upper hand in the management except in cases of rapid neurological deterioration in which surgical evacuation is required.

## Conclusion

Patients with aplastic anemia or any bleeding disorder conditions should be investigated thoroughly if symptoms denoted a CNS pathology. Concomitant cranial and spinal SDH rarely occurs, and more studies are advocated to be structured to study the specific pathophysiology and etiologies of this condition. Conservative management still has the upper hand. However, surgical intervention for evacuation may be required but in limited situations like progressive and severe neurological deterioration.

## Data Availability

All materials used in this article are owned by the authors, and/or no permissions are required.
